# Equal Expansion of Endogenous Transplant-Specific Regulatory T Cell and Recruitment Into the Allograft During Rejection and Tolerance

**DOI:** 10.3389/fimmu.2018.01385

**Published:** 2018-06-20

**Authors:** James S. Young, Dengping Yin, Augustin Georges Louis Vannier, Maria-Luisa Alegre, Anita S. Chong

**Affiliations:** ^1^Department of Surgery, The University of Chicago, Chicago, IL, United States; ^2^Department of Medicine, The University of Chicago, Chicago, IL, United States

**Keywords:** regulatory T cells, allospecific T cells, murine heart transplant, transplantation immunology, conventional T cells, transplant tolerance, immunosuppression, costimulatory blockade

## Abstract

Despite numerous advances in the definition of a role for regulatory T cells (Tregs) in facilitating experimental transplantation tolerance, and ongoing clinical trials for Treg-based therapies, critical issues related to the optimum dosage, antigen-specificity, and Treg-friendly adjunct immunosuppressants remain incompletely resolved. In this study, we used a tractable approach of MHC tetramers and flow cytometry to define the fate of conventional (Tconvs) and Tregs CD4^+^ T cells that recognize donor 2W antigens presented by I-A^b^ on donor and recipient antigen-presenting cells (APCs) in a mouse cardiac allograft transplant model. Our study shows that these endogenous, donor-reactive Tregs comparably accumulate in the spleens of recipients undergoing acute rejection or exhibiting costimulation blockade-induced tolerance. Importantly, this expansion was not detected when analyzing bulk splenic Tregs. Systemically, the distinguishing feature between tolerance and rejection was the inhibition of donor-reactive conventional T cell (Tconv) expansion in tolerance, translating into increased percentages of splenic FoxP3^+^ Tregs within the 2W:I-A^b^ CD4^+^ T cell subset compared to rejection (~35 vs. <5% in tolerance vs. rejection). We further observed that continuous administration of rapamycin, cyclosporine A, or CTLA4-Ig did not facilitate donor-specific Treg expansion, while all three drugs inhibited Tconv expansion. Finally, donor-specific Tregs accumulated comparably in rejecting tolerant allografts, whereas tolerant grafts harbored <10% of the donor-specific Tconv numbers observed in rejecting allografts. Thus, ~80% of 2W:I-A^b^ CD4^+^ T cells in tolerant allografts expressed FoxP3^+^ compared to ≤10% in rejecting allografts. A similar, albeit lesser, enrichment was observed with bulk graft-infiltrating CD4^+^ cells, where ~30% were FoxP3^+^ in tolerant allografts, compared to ≤10% in rejecting allografts. Finally, we assessed that the phenotype of 2W:I-A^b^ Tregs and observed that the percentages of cells expressing neuropilin-1 and CD73 were significantly higher in tolerance compared to rejection, suggesting that these Tregs may be functionally distinct. Collectively, the analysis of donor-reactive, but not of bulk, Tconvs and Tregs reveal a systemic signature of tolerance that is stable and congruent with the signature within tolerant allografts. Our data also underscore the importance of limiting Tconv expansion for high donor-specific Tregs:Tconv ratios to be successfully attained in transplantation tolerance.

## Introduction

Life-long pharmacological immunosuppression is necessary to prevent the rejection of allografts; however, side-effects, on-target toxicities, and high costs of drugs, together with emergent chronic allograft rejection, have prompted research toward inducing long-term graft acceptance following transient immunosuppressive therapy (1). Regulatory T cells (Tregs) that express the transcription factor FoxP3 (FoxP3^+^ Tregs) have been shown to be critical for the successful induction and maintenance of peripherally induced transplantation tolerance in many experimental models. Their importance has been inferred from observations of Treg accumulation in tolerant allografts (2–7), peripheral conversion of FoxP3^−^ CD4^+^ T [conventional T cell (Tconv)] cells into FoxP3^+^ Tregs under tolerance-inducing therapy (8, 9), and the inability to develop, as well as the reversal of, transplantation tolerance in recipients depleted of Tregs (6, 7, 10, 11). The sufficiency of Tregs to mitigate rejection, or facilitate transplantation tolerance, has been demonstrated by the adoptive transfer of FoxP3^+^-enriched T cells (12–15). Finally, donor-specificity and infectious tolerance are key features of transplantation tolerance, and the notion that donor-specific Tregs confer both specificity and infectious tolerance is supported by observations of superior efficacy of transferred allospecific Tregs over polyclonal Tregs at suppressing alloimmune responses (16–22). Allospecific Tregs for those studies were enriched by alloantigen-stimulated expansion *in vitro* or *in vivo*, and more recently, generated *via* engineered expression of alloantigen-reactive T cell receptors. While some caveats can be raised that experimental mouse models are highly reductionist and/or attenuated, observations made with these models have nevertheless provided the rationale for adoptive Treg therapy in transplantation (1).

Many different mechanisms have been implicated in the ability of Tregs to limit the Tconv responses in autoimmunity, infection, tumor immunity, and allogeneic transplantation [reviewed in Ref. (23)]. By virtue of constitutive expression of CD25, which can serve as an “IL-2 sink,” and of CTLA-4, which reduces costimulatory CD80 and CD86 signals from antigen-presenting cells (APC), Tregs diminish alloreactive T cell responses [reviewed in Ref. (24)]. Furthermore, activated Tregs can upregulate a number of suppressive mechanisms, including the production of IL-10, IL-35, TGF-β, ectoenzymes CD39 and CD73, as well as granzyme that functions to limit the expansion or function of Tconvs (24). Finally, Tregs can differentiate into specialized subsets that traffic to site of inflammation, where they preferentially suppress to select immune cell effector functions; e.g., Tbet^+^, IRF4^+^. Rorγt^+^, Bcl6^+^ Tregs inhibiting Th1, Th2, Th17, and Tfh responses, respectively [reviewed in Ref. (25)].

Following allograft transplantation, Tregs recognizing intact donor MHC or donor peptide presented on recipient MHC become activated and migrate into the allograft. Similar to Tconvs, alloreactive Tregs that recognize intact donor MHC molecules directly are present at ~100-fold higher frequency than Tregs that recognize donor-derived peptides presented indirectly on host MHC molecules (26). Observations that the combination of adoptively transferred indirect and direct alloreactive Tregs promoted better graft survival than each subset alone (12, 18, 22), have prompted Tang and Vincenti (1) to speculate that direct alloreactive Tregs are critical for the induction of tolerance, while Tregs with indirect alloantigen specificity are required for the maintenance of tolerance. Finally, a third population of tissue-resident Tregs that promote tissue repair may also accumulate into both rejecting and tolerant allografts, in response to an IL-33:ST2 axis rather than by TCR engagement (27–30). Indeed, early studies by Graca et al. (31) suggest that non-specific Tregs may contribute to tolerance, possibly through bystander effects.

The fate of endogenous Tregs with direct or indirect alloreactive specificity in acute rejection and tolerance is currently poorly characterized, as transplant studies analyzing endogenous Tregs have focused on bulk Tregs, of which only a small fraction is expected to be donor-reactive. We adapted the technique pioneered by Jenkins and colleagues (32, 33) that uses peptide:MHC tetramers to identify endogenous antigen-specific T cells, and applied it to track donor-reactive Tregs and Tconvs capable of recognizing a 2W (EAWGALANWAVDSA) donor-derived peptide presented by I-A^b^ expressed on both donor and recipient APCs. In naïve C57BL/6 mice, 2W:I-A^b^ tetramers specifically recognize a CD4^+^ T cell subset comprising ~7.5% Tregs that likely arose as a result of their recognition of cross-reactive self-epitopes (34). We observed that these donor-reactive Tregs expanded comparably in acute rejection and tolerance, resulting in similar absolute Treg numbers in the spleen and infiltrating heart allografts. The main distinguishing factor between rejection and tolerance was the markedly reduced expansion of donor-reactive Tconvs in tolerance. Thus, the greater donor-reactive Treg:Tconv ratios observed in tolerance compared to rejection in both the spleen and allograft were due to the control of Tconv expansion in tolerance. Importantly, these observations were not observed when bulk Tregs from the spleen were assessed. We further analyzed the impact of three distinct classes of immunosuppressive drugs on accumulation of graft-reactive Tregs and Tconvs and the phenotype of Tregs in rejection and tolerance, underscoring the utility of this approach for gaining new insights into the biology of transplant-specific Tregs and Tconvs in rejection and tolerance.

## Results

### Comparable Expansion of Donor-Specific Tregs in the Spleen of Tolerant and Rejecting Recipients

**Figure 1:**
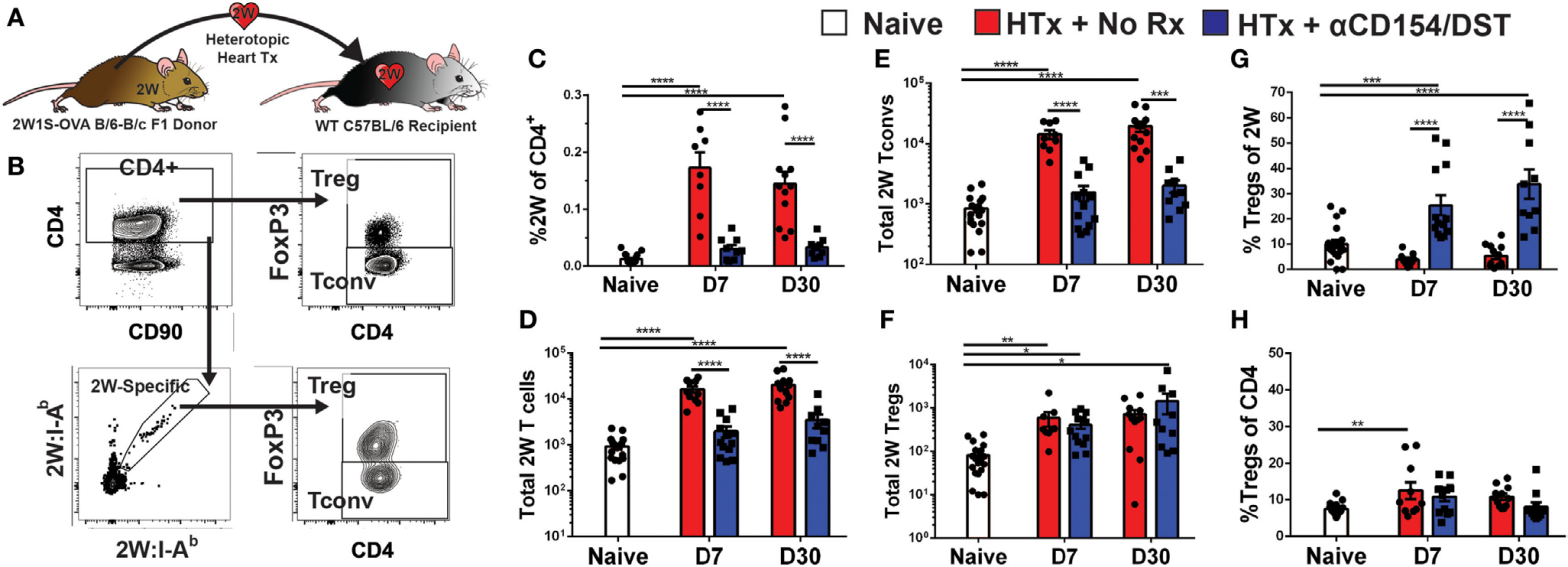
Increased donor-specific regulatory T cell (Treg) percentages in the spleens of costimulatory blockade-induced tolerant recipients is due to inhibition of conventional T cell (Tconv) expansion and modest accumulation of Tregs. C57BL/6 recipients were transplanted with heterotopic heart allografts from Act.2W-OVA^+^ BALB/c × C57BL/6 F1 donors. Recipients were either given anti-CD154 on days 0, 7, and 14 post-transplantation plus donor splenocyte infusion on day 0 (αCD154/DST), or were untreated (No Rx). On day 7 or day 30 post-transplantation, mice were sacrificed and their splenocytes were analyzed. **(A)** Cartoon depicting the experimental protocol. **(B)** Sample gating strategy of CD4^+^ splenocytes for 2W:I-A^b^ (2W) Tregs. **(C)** Percentage 2W-specific cells among CD4^+^ T cells. **(D)** Total number of 2W-specific CD4^+^ T cells in the spleen. **(E)** Total number of 2W-specific FoxP3^–^CD4^+^ Tconv in the spleen. **(F)** Total number of 2W-specific FoxP3^+^CD4^+^ Tregs in the spleen. **(G)** Percentage of FoxP3^+^ Tregs among 2W-specific CD4^+^ T cells. **(H)** Percentage of FoxP3^+^ Tregs among all splenic CD4^+^ T cells. **p < 0.01, ***p < 0.001, ****p < 0.0001 by one-way ANOVA when comparing between naïve and individual time points and by two-tailed *t*-test when comparing between treatment groups. Mean ± SEM is shown, and each point represents one animal from 4–5 replicate experiments per time point (*n* = 8–13).

We used an experimental system in which C57BL/6XBALB/c (F1) hearts expressing the 2W-OVA fusion protein, as a model antigen, were transplanted into C57BL/6 mice (Figure 1A). CD4^+^ cells recognizing the 2W peptide presented by I-A^b^ expressed by both donor and recipient APCs were identified using fluorescently labeled 2W:I-A^b^ tetramers. Importantly, these 2W:I-A^b^-reactive T cells represent a “tracer” population within a larger repertoire of donor-specific T cells recognizing incompatible MHC (H-2^d^) and minor BALB/c antigens, but are unlikely to be solely responsible for mediating rejection or tolerance in this fully mismatched transplant model. Following intracellular FoxP3 staining, the 2W:I-A^b^-binding Tregs (FoxP3^+^) and Tconvs (FoxP3^−^) from the spleen were assessed in untreated recipients that rejected their grafts in 10 ± 1 days, and in recipients treated at the time of transplantation with anti-CD154 and donor splenocytes and accepted their allografts long-term (Figure 1B; Figure S1 in Supplementary Material). In this tolerance model, we previously reported that T and B cell responses, measured by IFNγ and DSA production, respectively, are persistently curtailed (4, 10, 35–37).

As anticipated, we observed that rejection was associated with a significant accumulation of splenic 2W:I-A^b^-binding T cells that reached 0.2% of total CD4^+^ cells at days 7 and 30 post-transplantation, compared to ~0.01% in naïve mice (Figure 1C). This ~20-fold increase in 2W:I-A^b^-reactive T cells in rejecting animals was mainly due to the expansion of Tconvs, and a modest ~eightfold increase in 2W:I-A^b^-binding Tregs (Figures 1D–F). The unequal fold expansion between Tconvs and Tregs resulted in a net decrease in the percentage of Tregs among 2W:I-A^b^-reactive T cells (Figure 1G). In contrast, accumulation of splenic 2W:I-A^b^-reactive Tconvs was prevented in tolerant recipients relative to naïve mice (Figures 1D,E), while the expansion of 2W:I-A^b^-reactive Tregs was comparable in acute rejection and tolerance (Figure 1F). This modest increase in donor-specific Treg numbers nevertheless translated into a significant increase in 2W:I-A^b^-binding Treg percentages among 2W:I-A^b^-binding CD4^+^ T cells in the spleen of tolerant mice compared to naïve or acute rejection mice, both at days 7 and 30 post-transplantation (Figure 1G). Importantly, analysis of bulk Tregs and Tconvs from the spleen during acute rejection and tolerance failed to capture these differences in either total numbers or percentages (Figure S2 in Supplementary Material; Figure 1H), underscoring the importance of tracking donor-specific T cells. Thus, our data support the prediction of a significant difference in donor-specific Tconvs during rejection, and reveal that donor-specific Tregs behave comparably in cardiac allograft transplantation tolerance and rejection.

### Tolerance-Induced Expansion of Donor-Specific Tregs Is Inhibited by Rapamycin and Cyclosporine A (CsA)

**Figure 2:**
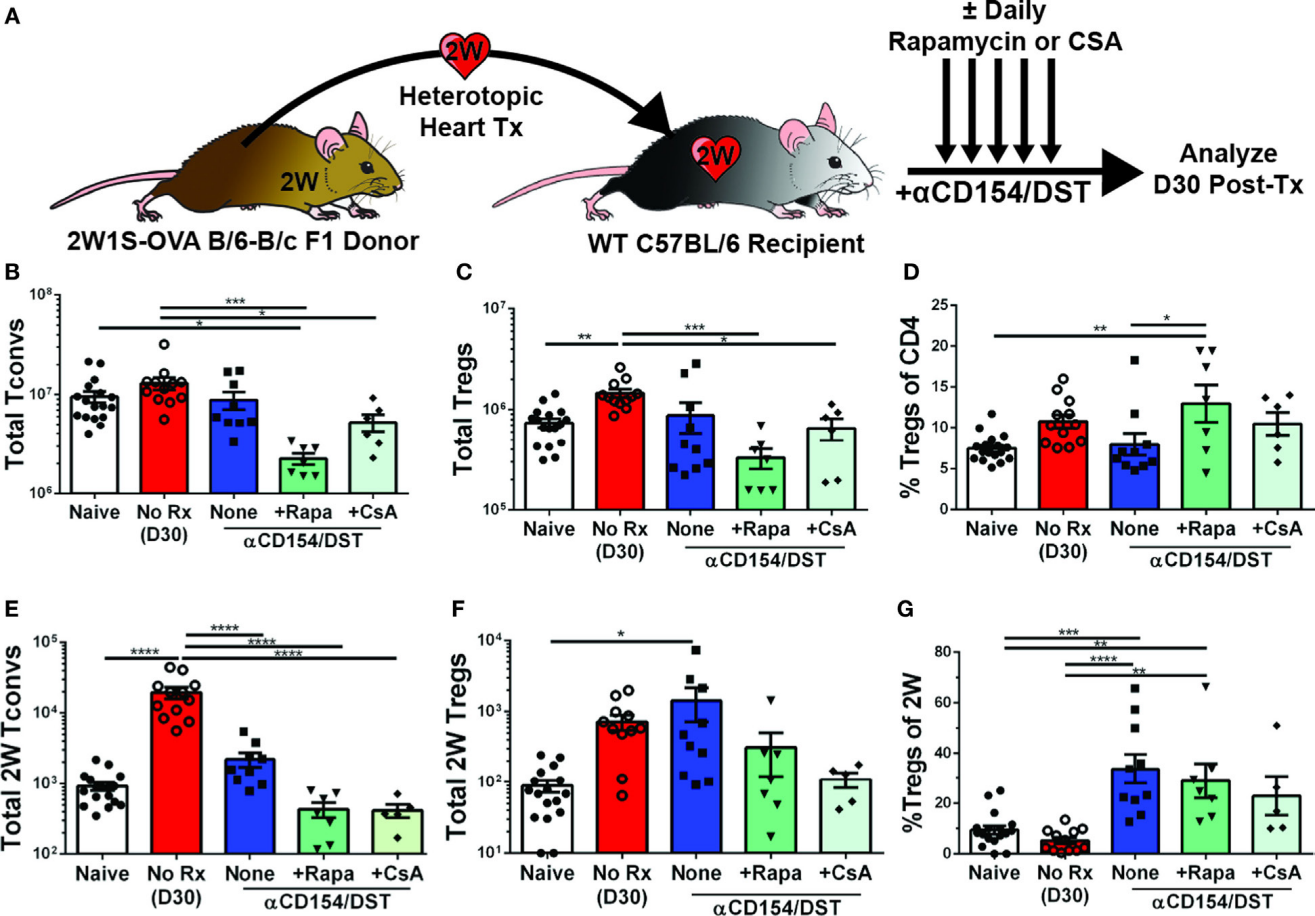
Rapamycin and cyclosporine A (CsA) inhibit expansion of allospecific conventional T cell (Tconv) but do not promote regulatory T cell (Treg) accumulation in costimulatory blockade-induced tolerant recipients. C57BL/6 recipients were transplanted with heterotopic heart allografts from Act.2W-OVA^+^ BALB/c × C57BL/6 F1 donors as described in Figure 1. Some recipients were untreated (No Rx), or treated with αCD154/DST also received daily injections of 2.5 mg/kg rapamycin or 50 mg/kg CsA, and all recipients were sacrificed on 30 days post-transplantation. Data for the naïve and anti-CD154/DST groups are from Figure 1. **(A)** Cartoon depicting experimental design. **(B)** Total splenic CD4^+^FoxP3^–^ Tconv and **(C)** CD4^+^FoxP3^+^ Tregs. **(D)** Percentage of FoxP3^+^ cells among CD4^+^ T splenocytes. **(E)** Splenic 2W:I-A^b^(2W)-specific Tconvs and **(F)** Tregs were enumerated. **(G)** Percentage of Tregs among 2W-specific CD4^+^ T splenocytes. *p < 0.01, **p < 0.01, ***p < 0.001, ****p < 0.0001 by one-way ANOVA. Mean ± SEM is shown, and each point represents one mouse from 3–4 replicate experiments (*n* = 7–19). The Naïve, No Rx, and αCD154/DST groups are from Figure 1.

Calcineurin inhibitors such as CsA are known to impair Treg activation, inhibit the generation of peripheral Tregs, and convert Tregs into Tconv by blocking FoxP3 mRNA expression, while mTOR inhibitors such as rapamycin are thought to promote Treg development, stability, and function by blocking the PI3K-Akt-mTOR signaling axis (38, 39). Because bulk Tregs and Tconvs were assessed in those studies while our investigations revealed that bulk Tregs do not predict the behavior of donor-specific Tregs (Figure 1), and because recent observations indicate that T cell receptor (TCR) signaling further regulates Treg cell differentiation, maintenance, and function [(40, 41); reviewed in Ref. (42)], we reasoned that it may be informative to reexamine the effects of CsA and rapamycin on donor-specific Tregs. To test whether these drugs had the ability to inhibit or promote donor-specific Tregs, mice that received the tolerogenic treatment of anti-CD154/DST also received daily treatment of rapamycin (2.5 mg/kg) or CsA (50 mg/kg). Splenic analyses were performed on day 30 post-transplantation when all the grafts were still beating (Figure 2; Figure S1A in Supplementary Material). Rapamycin significantly increased bulk Treg percentages (8% Tregs for anti-CD154/DST vs. 13% Tregs for anti-CD154/DST + rapamycin) by preferentially reducing bulk Tconv over bulk Treg numbers (Figures 2B–D). In the same mice, rapamycin treatment reduced the numbers of 2W:I-A^b^-binding Tconvs and Tregs comparably, thereby preserving the increase in 2W:I-A^b^-reactive Treg percentages observed with anti-CD154/DST treatment alone (Figures 2C–G). In contrast, the relatively high dose of CsA had no significant effect on bulk or 2W:I-A^b^-binding Treg percentages, due to a similar reduction in both Treg and Tconv numbers (Figures 2C–G). These results demonstrate that both rapamycin and CsA, at the doses used, profoundly inhibited donor-specific Tconvs compared to untreated controls and that neither drug promoted donor-reactive Treg expansion over that observed with anti-CD154/DST.

### Transient CTLA4-Ig Induces the Expansion, While Sustained CTLA4-Ig Depletes, Donor-Specific Tregs

**Figure 3:**
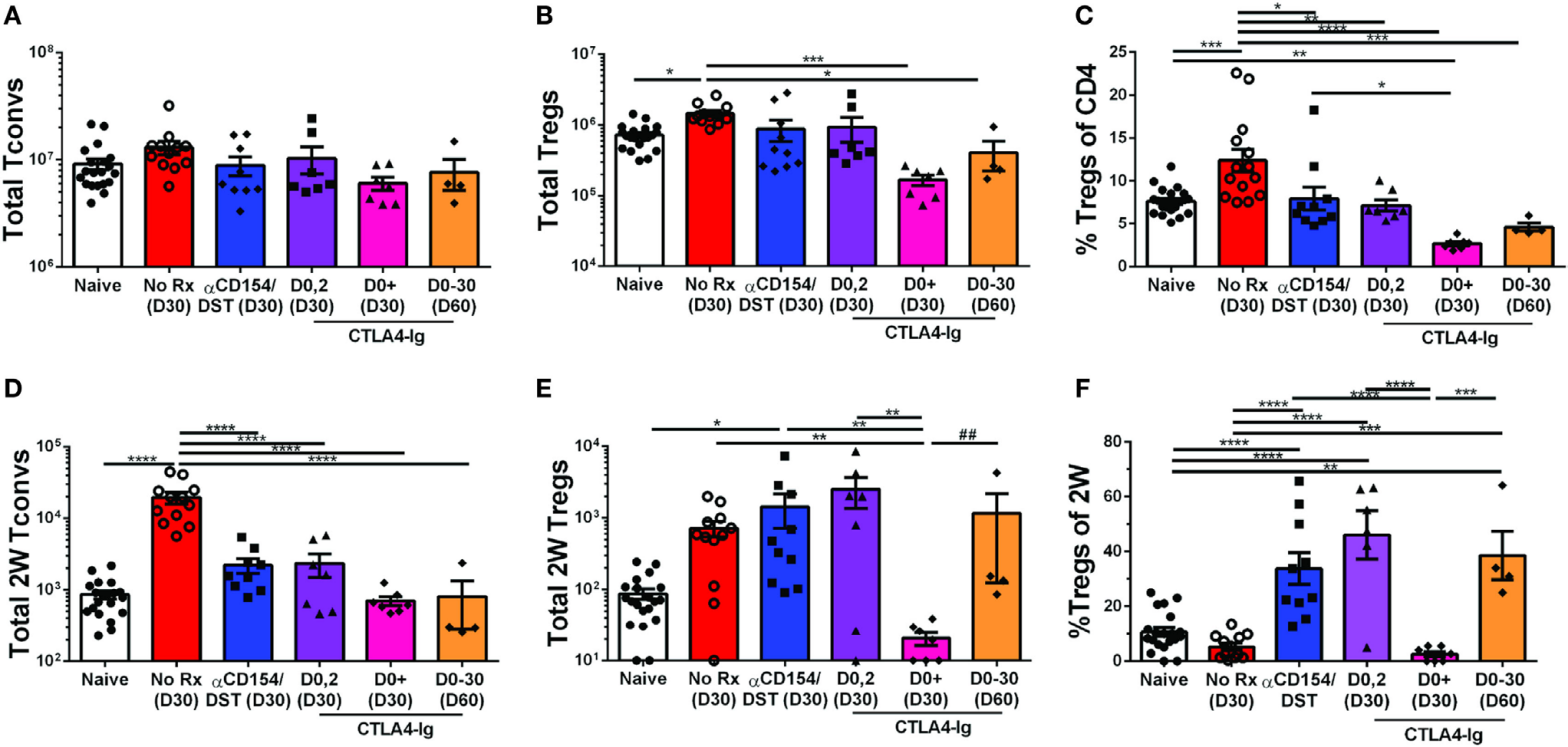
Divergent effect of transient vs. continuous CTLA4-Ig on allospecific conventional T cell (Tconv) and regulatory T cells (Tregs). C57BL/6 recipients were transplanted with heterotopic heart allografts from Act.2W-OVA^+^ BALB/c × C57BL/6 F1 donors. Recipients were untreated (No Rx), treated with anti-CD154/DST, CTLA4-Ig on days 0 and 2 post-transplantation, or twice a week from day 0 to day 30 post-transplantation (CTLA4-Ig D0^+^ and D0-30) and sacrificed at either D30 or D60 post-Tx (date of sacrifice in parenthesis). Data for naïve and 30 days post-transplantation anti-CD154/DST groups are from Figures 1 and 2. **(A)** Splenic CD4^+^FoxP3^–^ Tconv and **(B)** CD4^+^FoxP3^+^ Tregs were enumerated. **(C)** Percentage of FoxP3^+^ cells among CD4^+^ T splenocytes. **(D)** Splenic 2W:I-A^b^(2W)-specific Tconv and **(E)** 2W-specific Tregs were enumerated. **(F)** Percentage of FoxP3^+^ Tregs among 2W-specific CD4^+^ T splenocytes. *p < 0.01, **p < 0.01, ***p < 0.001, ****p < 0.0001 by one-way ANOVA, ^##^*p* < 0.01 by two-tailed ranked *t*-test. Mean ± SEM is shown, and each point represents one mouse from 2–4 replicate experiments (*n* = 4–19). The Naïve, No Rx, and αCD154/DST groups are from Figure 1.

The high-affinity CTLA4-Ig, belatacept, currently approved for use in kidney transplant recipients (43) inhibits CD28 costimulation, a critical pathway for Tconv cell activation as well as Treg cell development and homeostasis. However, a number of studies indicate that TCR engagement controls the expression of a large number of genes in activated Tregs required for suppressor function [(40, 41); reviewed in Ref. (42)], raising the possibility that the impact of CTLA4-Ig on donor-specific Tregs whose TCRs are engaged may be different from the effect of CTLA4-Ig on bulk Tregs. To address this possibility, we analyzed heart transplant recipients that received only 2 doses of CTLA4-Ig (0.5 mg/dose) or received CTLA4-Ig twice a week for 4 weeks. Additionally, we examined a small group of mice treated with CTLA4-Ig twice a week until D30 and then left untreated until D60. Mice were sacrificed and their spleens were harvested on D30 or D60 post-transplantation, when all the heart allograft were still beating except for one from the CTLA4-Ig D0-30 group was rejected by D60 (Figure S1B in Supplementary Material). Transient CTLA4-Ig had no significant impact on the total number of bulk Tregs and Tconvs, whereas continuous high-dose CTLA4-Ig significantly reduced bulk Treg percentages by preferentially depleting Tregs over Tconvs (Figures 3A–C). In contrast, analysis of 2W:I-A^b^-reactive T cells revealed that transient CTLA4-Ig treatment resulted in a tolerance profile that was similar to that observed with anti-CD154/DST treatment (Figures 3D–F), with 2W:I-A^b^-positive Tregs expanding modestly and Tconv expansion inhibited. Strikingly, continuous CTLA4-Ig treatment did not significantly reduce the numbers of 2W:I-A^b^-reactive Tconvs compared to naïve mice or transient CTLA4-Ig treatment, but significantly diminished 2W:I-A^b^-binding Treg numbers, resulting in donor-specific Treg percentages that were reduced compared to transient CTLA4-Ig-treated mice (Figures 3D–F). Thus, TCR-signaling does not override the deleterious effects of continuous high-dose CTLA4-Ig on Tregs, and graft acceptance is the result of inhibition of Tconv numbers and function by CTLA4-Ig, as previously demonstrated by Xin et al. (44). Interestingly, 2W:I-A^b^-reactive Treg numbers but not Tconv numbers, significantly recovered (Figures 3D–F), and 3 of 4 grafts survived for >30 days after weaning off CTLA4-Ig. (Figure S1C in Supplementary Material).

### Comparable Accumulation of Bulk and Donor-Specific Tregs in the Tolerant and Rejecting Allografts

**Figure 4:**
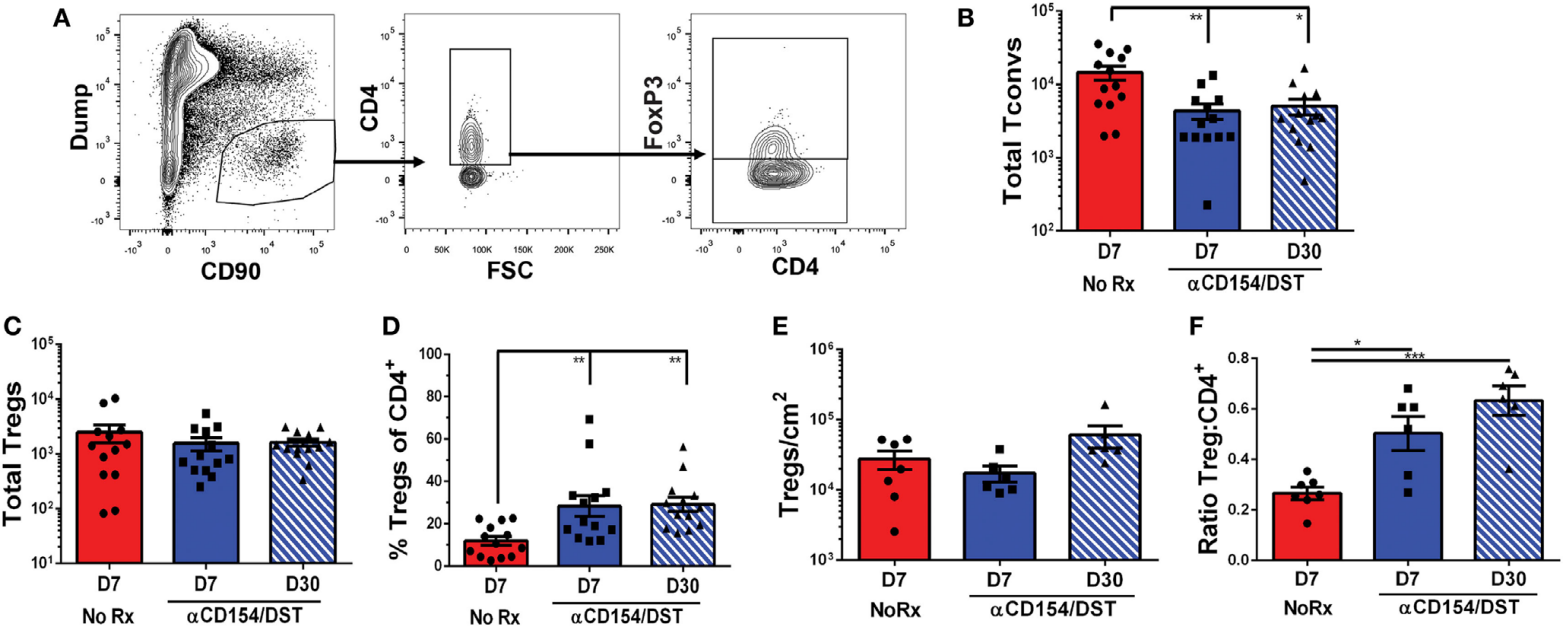
Regulatory T cells (Tregs) infiltrate comparably into allografts in rejection and tolerance while conventional T cell (Tconv) infiltration is reduced in tolerance. C57BL/6 recipients were as described in Figure 1. On day 7 or day 30 post-transplantation, mice were sacrificed and their grafts and graft-infiltrating cells were analyzed by flow cytometry **(A–D)** or immunohistochemistry **(E–F)**. **(A)** Gating strategy for graft-infiltrating CD4^+^ Tregs. **(B)** Total number of graft-infiltrating FoxP3^–^CD4^+^ Tconv and **(C)** FoxP3^+^CD4^+^ Tregs. **(D)** Percentage of FoxP3^+^ cells among CD4^+^ graft-infiltrating T cells. **(E)** Total number of FoxP3^+^ cells per cm^2^ from entire heart histology sections and **(F)** Ratio of FoxP3^+^:CD4^+^ cells from matched subsections of sequentially cut histology sections (four subsections at 10× magnification per mouse). *p < 0.01, **p < 0.01, ***p < 0.001 by one-way ANOVA. Mean ± SEM is shown, and each point represents one mouse from 7–8 replicate experiments (*n* = 13).

Donor-specific T cell quantification in the spleen informs on how Tconvs and Tregs accumulate systemically in response to graft-derived antigens, as well as on the impact of tolerance-inducing regimens or immunosuppression, but ultimately whether a graft is rejected or accepted depends on events within the allograft. We, therefore, examined the accumulation of total CD4^+^ Tconvs and Tregs in F1 heart allografts undergoing rejection on D7 post-transplantation or those destined to be tolerant on D30 post-transplantation (Figure 4A). On D7 post-transplantation, the total numbers of CD4^+^ Tconvs in the rejecting allografts were threefold higher than in tolerant allografts (Figure 4B). Consistent with responses in the spleen, the numbers of Tregs infiltrating the grafts were not significantly different between the rejecting and tolerant groups on D7 post-transplantation (Figure 4C). This translated into an increase in the percentage of bulk Tregs in tolerant compared to rejecting allografts that persisted to D30 post-transplantation when the levels of circulating anti-CD154 had waned (Figure 4D).

**Figure 5:**
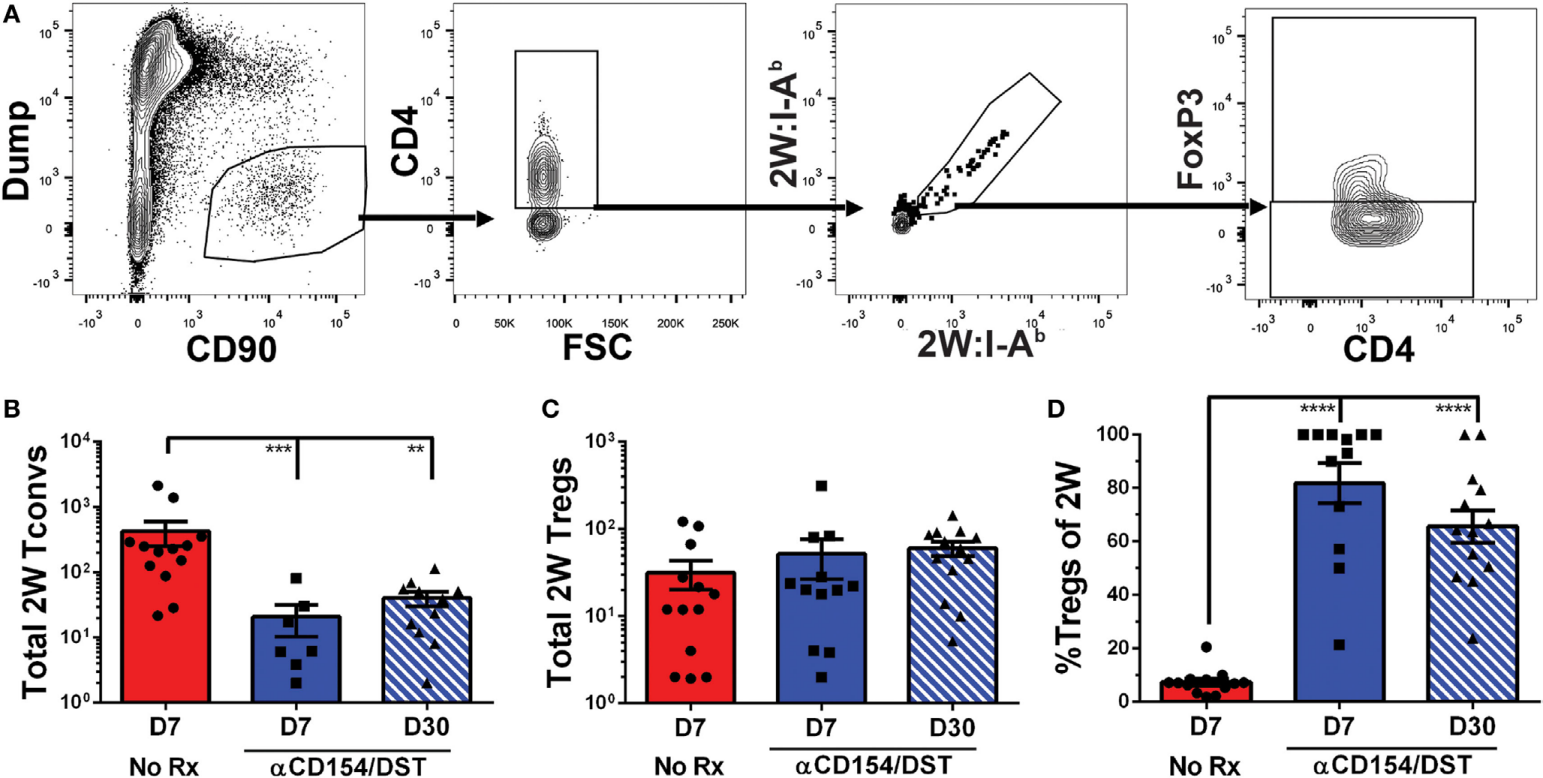
Donor-specific 2W:I-A^b^ regulatory T cells (Tregs) infiltrate comparably into allografts in rejection and tolerance while 2W:I-A^b^ conventional T cell (Tconv) infiltration is reduced in tolerance. Graft-infiltrating cells were analyzed from recipients as described in Figure 4. **(A)** Gating strategy for 2W:I-A^b^(2W)-specific FoxP3^+^CD4^+^ graft-infiltrating Tregs. **(B)** Graft-infiltrating 2W-specific CD4^+^FoxP3^–^ Tconv and **(C)** 2W-specific FoxP3^+^ Tregs were enumerated. **(D)** Percentage of FoxP3^+^ cells among graft-infiltrating 2W-specific CD4^+^ T cells. **p < 0.01, ***p < 0.001, ****p < 0.0001 by one-way ANOVA. Mean ± SEM is shown, and each point represents one animal from 7–8 replicate experiments (*n* = 12–13).

In studies examining tissue-resident CD8^+^ memory cells, Steinert et al. (45) raised the possibility that lymphocyte isolation fails to recover most cells and biases against certain subsets. With those concerns in mind, we performed immunohistochemistry as an unbiased approach to identify all graft-infiltrating T cells. These analyses confirmed that the numbers of Tregs infiltrating tolerant vs. acutely rejecting allografts were comparable while the ratios of Tregs:CD4^+^ T cells were significantly elevated in tolerance compared to rejection (Figures 4E,F; Figure S3 in Supplementary Material). Following this validation, we went on to determine the rate of infiltration of 2W:I-A^b^-binding Tconvs and Tregs in tolerant (D7 & D30) and rejecting (D7) allografts (Figure 5; Figure S4 in Supplementary Material). Overall, there was a trend toward enrichment of 2W:I-A^b^-reactive T cells relative to total CD4^+^ T cells infiltrating rejecting allografts, and of 2W:I-A^b^-reactive Tregs relative to total Tregs infiltrating tolerant allografts (Figure S3 in Supplementary Material). Total numbers of donor-specific Tconvs were significantly reduced in tolerant grafts compared to rejecting allografts (Figure 5B), whereas the total numbers of Tregs were comparable (Figure 5C), resulting in reduced donor-specific Treg percentages in rejection and an increase to 60–80% of 2W:I-A^b^-reactive T cells being Tregs in tolerant allografts on D30 post-transplantation. These observations lead us to speculate that the majority of the non-2W:I-A^b^ graft-infiltrating CD4^+^ T cells are likely to be donor-reactive, in contrast to the spleen, where the majority of CD4^+^ T cells are non-donor reactive.

### Phenotypes of Tregs in the Tolerant and Rejecting Recipients

**Figure 6:**
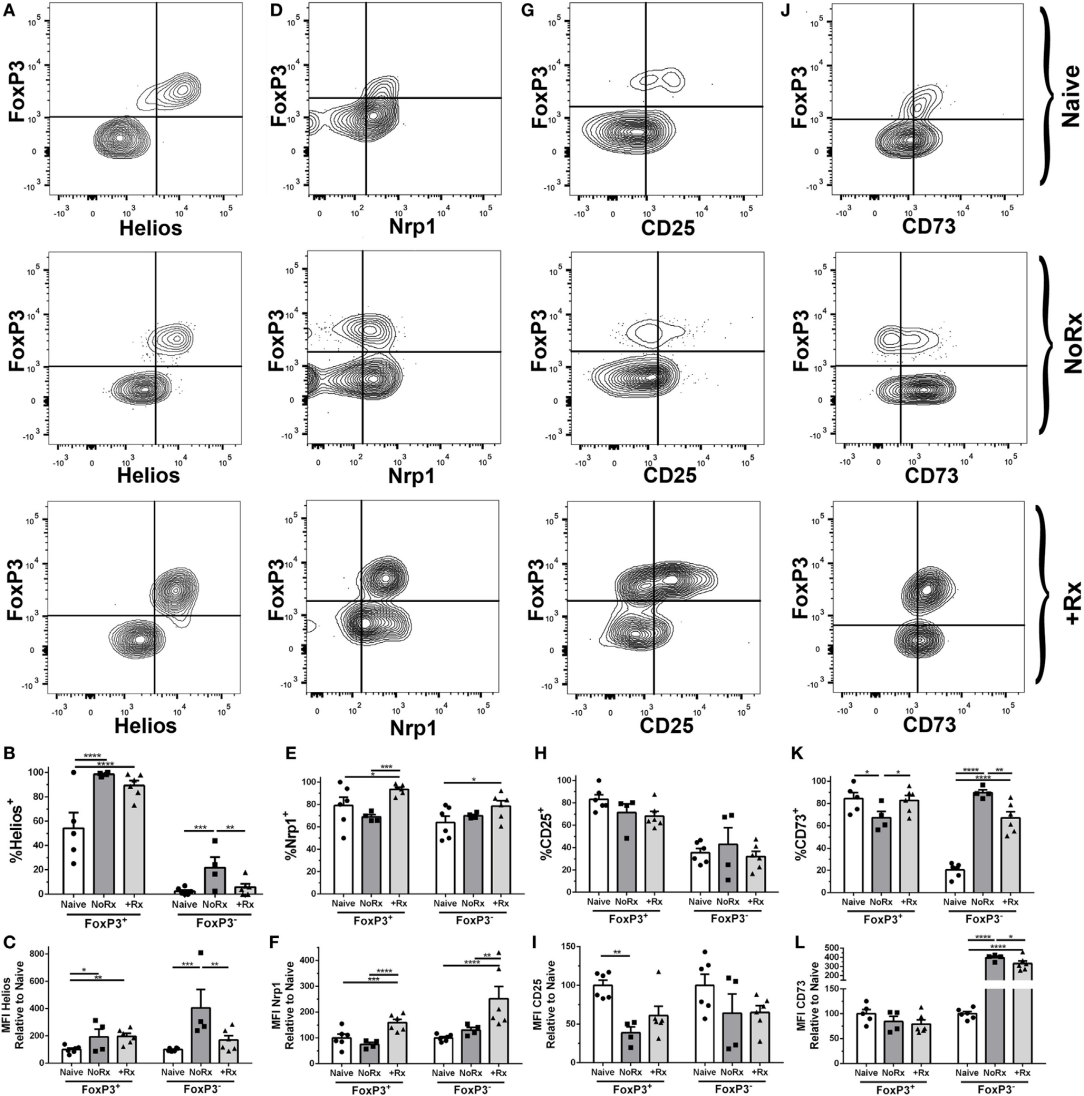
Phenotypic analysis of donor-specific 2W:I-A^b^ regulatory T cells (Tregs) isolated from recipients with rejecting and tolerant allografts at day 7 post-transplantation. C57BL/6 recipients transplanted with heterotopic heart allografts from Act.2W-OVA^+^ BALB/c × C57BL/6 F1 donors, were untreated (No Rx) or treated with αCD154/DST, and sacrificed on day 7 post-transplantation. Sample gating strategies **(A,D,G,J)**, percentage of cells positive **(B,E,H,K)**, and mean fluorescent intensity **(C,F,I,L)** relative to naïve 2W:I-A^b^-specific FoxP3^+^ or FoxP3^−^ cells of **(A–C)**, Helios; **(D–F)**, Neuropilin-1; **(G–I)**, CD25; and **(H–L)**, CD73; in naïve, acutely rejecting (No Rx), and αCD154/DST-treated (+Rx) animals. *p < 0.05, **p < 0.01, ***p < 0.001, ****p < 0.0001 by two-way ANOVA. Mean ± SEM is shown, and each point represents one animal from three replicate experiments (*n* = 4–6/group).

The equal numbers of Tregs in both tolerant and rejecting recipients prompted us to test whether these cells have distinct phenotypes. While the contribution of thymic-derived Treg expansion vs. induction cannot be definitively ascertained by phenotypic markers, Helios and Neuropilin-1 have been used in some studies to distinguish thymic-derived Tregs from peripherally induced Tregs (46–48). We observed increased expression of Helios on 2W:I-A^b^ Tregs from acutely rejecting and anti-CD154/DST-treated recipients, examined on day 7 post-transplantation, compared to naïve (Figures 6A–C). In contrast, the expression of Neuropilin-1 was significantly reduced on 2W:I-A^b^ Tregs in acute rejection compared to anti-CD154/DST-treated recipients (Figures 6D–F). Both CD25 and the ectoenzyme CD73 have been implicated in Treg function, by depriving Tconvs of IL-2 and through cyclic AMP-mediated inhibition (24). Unexpectedly, the expression of CD25 (MFI relative to naïve) was comparably reduced on 2W:I-A^b^ Tregs from rejecting and tolerant recipients (Figures 6G–I), but with a non-significant but similar trend in reduced percentage of Tregs that are CD25^+^. In contrast, the percentage of CD73^+^ 2W:I-A^b^ Tregs was modestly but significantly reduced in rejection, but not in tolerance, compared to naïve; however, there was no significant difference in the relative MFI between all three groups (Figures 6J–L). Notably, there was no significant MFI difference for bulk Tregs for all these markers (Figure S5 in Supplementary Material). Collectively, these data suggest that donor-specific Tregs may have distinct functionality in naïve, rejecting, and tolerant recipients.

We also compared the expression of Helios, Neuropilin-1, CD25, and CD73 on 2W:I-A^b^ Tconvs (Figure 6). Helios was upregulated on Tconvs only in rejection (Figures 6A–C), while Nrp-1 was significantly upregulated on Tconvs only in tolerance (Figures 6D–F). These results are consistent with Helios being a marker of Tconv activation, and of Nrp-1 downregulation upon TCR activation on Tconvs (49–51). CD25 (MFI relative to naïve) was downregulated, whereas the ectoenzyme CD73 was significantly upregulated, in both rejection and tolerance (Figures 6G–I,J–L). The differences in Helios and Neuropilin-1 expression on Tconvs in tolerance and rejection prompted us to further investigate their relative proliferation capacity, by staining for Ki67 (Figure S6 in Supplementary Material). The lower percentage of Ki67^+^ cells among the 2W:I-A^b^ Tconvs in tolerance compared to rejection is consistent with their reduced numbers observed at both day 7 and day 30 post-transplantation.

## Discussion

Regulatory T cells have been implicated in the induction and maintenance of donor-specific transplantation tolerance. Using a powerful new approach to track the fate of donor-specific Tconvs and Tregs in both the periphery and the allografts, we show that endogenous polyclonal CD4^+^ T cells recognizing a single donor-derived antigen can exist as Tregs or Tconvs prior to transplantation, and undergo different expansion profiles during acute rejection and tolerance. Specifically, we show that the increased percentages of FoxP3^+^ Tregs within the 2W:I-A^b^-reactive CD4^+^ T cell subset in spleen (~35 vs. <5%) and allograft (~80 vs. ≤10%) observed in tolerance is due to a modest increase in Tregs and inhibition of Tconvs accumulation, while the decreased percentages of donor-specific Tregs in rejection is due to the same modest increase in Tregs and a ~log increase in Tconvs numbers. Since these changes were observed both in the spleen/lymph nodes as well as in the graft, we conclude that the lack of accumulation of Tconvs in the tolerant graft was most likely due to the lack of expansion. These insights gained from the analysis of donor-reactive Tregs and Tconvs from the spleen and allograft underscores the importance of limiting Tconv expansion to facilitate achieving high Treg:Tconv ratios, and is consistent with the hypothesis put forward by Tang and colleagues (52) that a high Treg to Tconv ratio is needed both systemically and in the graft to control rejection and promote tolerance. If Tconv expansion is not controlled, it would be extremely challenging to achieve these high Treg:Tconv ratios in the lymph node, spleen, and allograft, and for T cell-mediated rejection to be held in check. We acknowledge limitations of our approach in that we only analyzed donor-specific Tconvs and FoxP3^+^ Tregs to one single model antigen, and it will be important to test whether the same rules apply to T cells directly recognizing intact allogeneic MHC or other donor antigens presented indirectly by recipient APCs, and also to non-FoxP3 Tregs and B cells (53–60). Indeed the differential fates of T cells with direct vs. indirect alloreactivity has been previously reported (61). Finally, how TCR affinity and antigen abundance affect the fate of Tconv and Tregs requires further investigation.

The well-documented Treg-promoting property of rapamycin has been exploited for manufacturing Tregs *in vitro* (38), and raises the possibility that it may also promote the *in vivo* development of tolerance by facilitating Treg expansion or conversion. Indeed, Gao et al. (62) reported that the pro-tolerogenic effects of rapamycin (3 mg/kg) could be explained by its ability to promote the conversion of Tconvs to Tregs, whereas CsA (20 mg/kg) did not have such properties. In contrast, Wang et al. (63) reported that rapamycin (1.25 mg/kg daily) comparably inhibited Treg and Tconv proliferation and promoted their apoptosis, resulting in no change in the percentages of Tregs among CD4^+^ cells. In addition, they reported no evidence of conversion of Tconvs into Tregs, and taken together, their studies did not support a net salutary effect of rapamycin on Tregs *in vivo*. We observed that both rapamycin and CsA, at the doses used, profoundly inhibited donor-specific Tconvs but neither drug promoted donor-reactive Treg expansion over that observed with anti-CD154/DST. Thus, our results are in contrast with previous reports of the damaging effects of calcineurin inhibitors and salutary effects of rapamycin on bulk Tregs, but are consistent with the findings of Wang et al. (63) of no net salutary effect of rapamycin on Tregs. These observations suggest a cautious approach to the use of these immunosuppressants during tolerance induction.

CD28 is non-redundant for Tregs, as CD28-deficient mice have reduced Treg numbers and develop exacerbated autoimmune diseases (64), suggests a potential deleterious effect of CTLA4-Ig. However, the demonstration that effector T cell activation requires higher CD80 and CD86 expression than is needed for maintaining Treg homeostasis, and that partial CD80 and CD86 blockade prevents the emergence of effector T cells while permitting Treg homeostasis in mouse models and in kidney transplant patients, has provided an explanation for the net immunosuppressive effects of CTLA4-Ig despite its effects on bulk Tregs (65, 66). We showed that transient CTLA4-Ig had distinct effects compared to continued CTLA4-Ig treatment; transient CTLA4-Ig resulted in a donor-specific Tconv and Treg numbers and percentages that were similar to anti-CD154/DST, whereas continuous CTLA4-Ig depleted donor-specific Tregs preferentially over Tconvs. We observed a rebound in donor-specific Tregs but not Tconvs and sustained graft acceptance upon weaning from continuous CTLA4-Ig treatment; an observation that bodes well for weaning trials of patients on Belatacept. We acknowledge the caveat that the immunosuppression used in this study does not accurately reflect the clinical scenario, where tacrolimus is the favored calcineurin inhibitor and the higher affinity Belatacept is used. Furthermore, the dosing and pharmacokinetics of these drugs in mice and humans are likely to be different, so extrapolation of our results to the clinical scenario should proceed cautiously with these issues in mind.

Our observations that bulk Tregs and Tconvs parallel donor-specific Treg and Tconvs within the allograft suggest that a simpler analysis of bulk Treg:Tconv ratios within the graft, either by immunohistochemistry or flow cytometry, may be sufficient to predict graft outcome rather than the more technically challenging analysis of donor-specific T cells. These observations of high ratios of bulk or endogenous donor-specific Tregs:Tconv infiltrating the tolerant allograft compared to the rejecting allograft are congruent with Fan et al. (8), who reported on adoptively transferred “color-coded” bulk Treg and Tconvs cells in islet allograft transplantation. We further observed that splenic bulk Tregs and Tconvs did not undergo the same changes in ratios or numbers as splenic donor-specific Treg and Tconv, which we speculate is due to changes in donor-reactive T cell frequencies being overshadowed by the absence of change in the vast majority of splenic T cells that are not graft-reactive. In contrast, Fan et al. (8) reported detectable differences in circulating Treg/Tconv ratios in the ear artery of tolerant vs. rejecting recipients, and raised the possibility that peripheral blood analysis may be useful for the diagnosis of tolerance induction. Taken together, the MHC tetramer-based *ex vivo* tracking of endogenous donor-specific Tregs and Tconvs is a tractable approach that complements the more technically challenging use of adoptively transferred fluorescently tagged Tregs and Tconvs and *in vivo* confocal microscopy or *in vivo* flow cytometry reported by Fan et al. (8), for gaining insights into the cellular response in allografts in the spleens of allograft recipients.

The comparable, albeit modest, increase in donor-specific Treg numbers in tolerance and rejection suggest that the inflammatory conditions associated with acute allograft rejection did not reduce the rate of Treg accumulation, and that the expansion of donor-specific Tregs is not inhibited by anti-CD154. The latter observations are consistent with the findings by Jarvinen et al. (67) that loss of CD154 expression on Tregs did not prevent skin allograft acceptance, and that CD154 is expressed on a majority of *in vitro* activated Tconvs but only on 4–9% of activated Tregs (68). The observation that equal numbers of donor-specific Tregs accumulate in tolerance and rejection raises the possibility that the contribution of natural vs. induced Tregs, and the function of Tregs, in acute rejection and tolerance may be different. In acute rejection but not in tolerance, alloreactive Tconv cells acquire the ability to produce proinflammatory cytokines that may reduce the suppressive function of FoxP3^+^ Tregs. For instance, Treg frequencies in patients with rheumatoid arthritis or colitis were elevated compared to healthy controls but their migratory capacity and ability to suppress were impaired (69–71). Conversely, others have reported that Tregs respond to inflammation by sharply increasing their suppressive function that then returns to baseline over time (72, 73). Thus the conflicting fates of Tregs under inflammatory conditions emphasized the need to elucidate the function of donor-specific Tregs in rejection and tolerance.

Because their low numbers preclude *ex vivo* functional analyses of 2W:I-A^b^ Tregs and Tconvs in rejection vs. tolerance, we compared their expression of Helios, Neuropilin-1, CD25, and CD73. Helios was upregulated on 2W:I-A^b^ Tregs from acutely rejecting and tolerant recipients compared to naïve; whereas Helios was only upregulated in the 2W:I-A^b^ Tconv during rejection. Neuropilin-1 was upregulated on 2W:I-A^b^ Tregs and Tconvs only in tolerance. Helios and Neuropilin-1 has been implicated as a marker of thymically derived Tregs (46–48), while Neuropilin-1, a receptor for TGFβ-1, plays a role in inducing a transcriptome that promotes Treg cell stability and function at inflammatory sites (74, 75). In addition, Helios has also been reported to be a marker of Tconv activation (49, 51). Thus it is unclear, whether the increase in percentage of Helios^+^, Neuropilin-1^+^ Tregs in tolerance is the result of expanded natural Tregs or whether these markers are upregulated on both natural and induced Tregs during tolerance induction. Furthermore, both CD25 and CD73 have been implicated in the function of Tregs, by depletion of IL-2 and by producing extracellular adenosine that curtails T cell function *via* adenosine receptor signaling (76, 77). The percentages of 2W:I-A^b^ Tregs expressing CD25 and CD73 in tolerant recipients were comparable to naïve, whereas CD73 was upregulated on 2W:I-A^b^ Tregs in Tconvs in rejection and tolerance. These data collectively suggest that 2W:I-A^b^ Tregs exhibit similarities but may also have modest differences in function in rejection and tolerance. In contrast, 2W:I-A^b^ Tconvs have reduced activation (Helios) and proliferation capacity in tolerance compared to rejection. This is likely to be due to anti-CD154 treatment, although it is possible that donor-specific or non-specific Tregs may also play a contributory role. We acknowledge limitations to this phenotypic approach, and that future delineation of Treg gene signatures using single cell RNA sequencing technologies is necessary for a deeper understanding of how endogenous donor-reactive Tregs respond to allografts under different inflammatory, immunosuppressive, or tolerance regimens. In addition, a more extensive investigation is ongoing to determine whether the persisting donor-specific Tconvs are curtailed by donor-specific or non-specific Tregs or by the acquisition of cell-intrinsic dysfunction. Such mechanistic insights may lead to the identification of new ways to induce graft acceptance, and may be applicable to understanding how antigen-specific Tregs respond in autoimmunity, tumor immunity, and infection.

## Materials and Methods

### Mice

C57BL/6 and BALB/c mice were purchased from Harlan Laboratories (Madison, WI, USA). 2W-OVA.Act^+^ C57BL/6-BALB/c F1 donors were bred from 2W-OVA.Act^+^ C57BL/6 males and BALB/c females and were screened to ensure expression of the transgene prior to use. Donor mice were 6–12 weeks of age at time of transplant. Recipients were 10–12 weeks of age at time of transplant. All animal experiments were approved by the Institutional Animal Care and Use Committee at the University of Chicago, and adhered to the standards of the NIH Guide for the Care and Use of Laboratory Animals.

### Heart Transplantation

Heterotopic heart transplantation was performed as previously described (10) by removing hearts from 2W-OVA.Act^+^ C57BL/6-BALB/c F1 donors and suturing the aorta and vena cava to the inferior vena cava in the abdomen of recipients. Ischemia time was less than 1 h for each heart.

### Tolerance Induction and Immunosuppression

Tolerance was primarily induced with a combination of anti-CD154 (MR1) at a dose of 500 μg/250 μg/250 μg on days 0, 7, and 14 post-transplantation in combination with 20 × 10^6^ donor splenocytes on day 0. Injections were performed intravenously on day 0 and intraperitoneally on days 7 and 14. CTLA4-Ig (Abatacept, Bristol-Myers Squibb, New York, NY, USA) was injected at a dose of 1 mg intravenously on day 0 post-transplantation and 500 µg intraperitoneally thereafter, either on day 2 post-transplantation or twice per week post-transplantation until the experimental endpoint. Rapamycin (Pfizer, New York, NY, USA) was prepared in a stock solution of 100% ethanol and diluted in 5% dextrose prior to intraperitoneal injection at a dose of 2.5 mg/kg (50 µg/animal). CsA (Sigma-Aldrich, St. Louis, MO, USA) was prepared in a stock solution of ethanol and castor oil and was diluted in 5% dextrose prior to intraperitoneal injection at a dose of 50 mg/kg (1 mg/animal).

### Tissue Harvesting and Histology

Spleens were harvested and passed through a 70 µM strainer and lysed in ACK lysis buffer (Quality Biological, Gaithersburg, MD, USA) for 2 min prior to analysis. Heart tissue for flow cytometry analysis was cut into approximately 2 mm^3^ pieces in HBSS (Gibco, Gaithersburg, MD, USA), incubated for 20 min at 37°C with collagenase II (Sigma-Aldrich, St. Louis, MO, USA), DnaseI (Roche, Branford, CT, USA), and HEPES (Gibco, Gaithersburg, MD, USA) prior to passing through a 70 µM strainer. Heart tissue for histology was fixed in 10% formalin for approximately 48 h prior to transfer to 70% ethanol for storage. Tissue was embedded in paraffin and then sequential cuts were made for H&E staining, and immunohistochemistry stains for CD4 and FoxP3.

Slides were scanned at 20× magnification using Aperio Slide Scanner (Leica Biosystems, Buffalo Grove, IL, USA), and representative or whole sections were sampled and the total number or percentage CD4^+^ and FoxP3^+^ cells were manually quantified in a single blind manner.

### Flow Cytometry

Samples were prepared with approximately 10^7^ cells per tube. In order to maximize rare cell numbers, some samples were enriched for CD4^+^ T cells via negative selection using magnetic beads (Miltenyi Biotec, Bergisch Gladbach, Germany) prior to staining. Samples were first incubated with 2W:I-A^b^ tetramer for 30 min at room temperature prior to the addition of other extracellular antibody-fluorochrome conjugates, with an additional 30 min of staining with no wash step in between. Samples were then washed with 2% FBS and fixed (Thermo Fisher, Waltham, MA, USA) for 1 h, washed in permeabilization buffer (Thermo Fisher, Waltham, MA, USA) and incubated overnight with intracellular staining antibodies. Samples were run on an LSR-II 4-12 flow cytometer (BD Bioscience, Woburn, MA, USA) and data were analyzed using FlowJo software (FlowJo, LLC, Ashland, OR, USA).

### Antibodies and Tetramers

CD49b-eFluor450 (Thermo Fisher, Waltham, MA, USA, Clone DX5, Cat #485971-82), CD11b-eFluor450 (Biolegend, Dedham, MA, USA, Clone M1/70, Cat #101224), CD11c-eFluor450 (Thermo Fisher, Waltham, MA, USA, Clone N418, Cat #48-0114-82), NK1.1-eFluor450 (Thermo Fisher, Waltham, MA, USA, Clone PK136, Cat #48-5941-82), Ter-119-eFluor450 (Thermo Fisher, Waltham, MA, USA, Clone Ter-119, Cat #48-5921-82), F4/80-eFluor450 (Thermo Fisher, Waltham, MA, USA, Clone BM8, Cat #48-4801-82), CD19-eFluor450 (Thermo Fisher, Waltham, MA, USA, Clone eBio1D3, Cat #48-0193-82), CD8a-eFluor450 (Thermo Fisher, Waltham, MA, USA, Clone 53-6.7, Cat #48-0081-82), CD8a-APC-Cy7 (Thermo Fisher, Waltham, MA, USA, Clone 53-6.7, Cat #47-0081-82), CD8a-BV605 (Biolegend, Dedham, MA, USA, Clone 53-6.7, Cat #100744), CD8a-PE-Cy7 (BD Bioscience, Woburn, MA, USA, Clone 53-6.7, Cat #552877), CD4-BV510 (BD Bioscience, Woburn, MA, USA, Clone RM4-5, Cat #563106), CD4-FITC (Thermo Fisher, Waltham, MA, USA, Clone RM4-5, Cat #11-0042-85), CD4-APC-Cy7 (Biolegend, Dedham, MA, USA, Clone GK1.5, Cat #100414), CD44-BV510 (BD Bioscience, Woburn, MA, USA, Clone IM7, Cat #563114), CD44-BV605 (Biolegend, Dedham, MA, USA, Clone IM7, Cat #103047), FoxP3-AlexaFluor488 (Thermo Fisher, Waltham, MA, USA, Clone FJK-16s, Cat #53-5773-82), FoxP3-PerCP-Cy5.5 (Thermo Fisher, Waltham, MA, USA, Clone FJK-16s, Cat #45-5773-82), CD90.2-APC-eFluor780 (Thermo Fisher, Waltham, MA, USA, Clone 53-2.1, Cat #470902-82), CD45.1-Percp-Cy5.5 (Thermo Fisher, Waltham, MA, USA, Clone A20, Cat #45-0453-82), CD45.2-PE-Cy7 (Biolegend, Dedham, MA, USA, Clone 104, Cat #109-830), CD25-PECy7 (Biolegend, Dedham, MA, USA, Clone 3C7, Cat #101915), CD73-PerCP/Cy5.5 (Biolegend, Dedham, MA, USA, Clone Ty/11.8, Cat #127213), Helios-PECy7 (Biolegend, Dedham, MA, USA, Clone 22F6, Cat #137235), Nrp1-PerCP/Cy5.5 (Biolegend, Dedham, MA, USA, Clone 3E12, Cat #145207), Ki67-PECy7 (BD Bioscience, Woburn, MA, USA, Clone B56, Cat #561283), 2W:I-A^b^ Tetramer-PE (peptide EAWGALANWAVDSA, National Institutes of Health Tetramer Core Facility, Atlanta, GA, USA, Cat #35240), and 2W:I-A^b^ Tetramer-APC (peptide EAWGALANWAVDSA, National Institutes of Health Tetramer Core Facility, Atlanta, GA, USA, Cat #35238).

### Statistical Analysis

Statistical analysis was performed using GraphPad Prism Software (GraphPad Software, Inc., La Jolla, CA, USA). For analyses involving more than two groups, a one-way ANOVA was performed with the Tukey correction. When comparing two groups at individual time points, multiple two-way *t*-tests were performed, with a Holm–Sidak correction.

## Ethics Statement

All animal experiments were approved by the Institutional Animal Care and Use Committee at the University of Chicago, and adhered to the standards of the NIH Guide for the Care and Use of Laboratory Animals.

## Author Contributions

JY performed the experiments, analyzed the data, and generated the figures, assisted in writing the manuscript. DY performed all the heart transplants. AV assisted in the experiments and data acquisition. M-LA participated in the research design and editing the manuscript. AC conceived and designed the research studies and wrote the manuscript.

## Conflict of Interest Statement

The authors declare that the research was conducted in the absence of any commercial or financial relationships that could be construed as a potential conflict of interest.

## Supplementary Material

Figure S1 Graft survival with costimulatory blockade and immunosuppression. Act.2W-OVA^+^ C57BL/6-BALB/c F1 hearts were transplanted into wild-type C57BL/6 recipients: **(A)** with no treatment (No Rx), or anti-CD154 on day 0, 7, and 14 with DST on day 0 (αCD154/DST) alone or in combination with daily intraperitoneal injections of rapamycin or cyclosporine A for 30 days. **(B)** With no treatment (No Rx), or CTLA4-Ig on days 0 and 2 post-transplant or twice a week starting on day 0 for 30 days, **(C)** with anti-CD154 on day 0, 7, and 14 with DST on day 0 or CTLA4-Ig twice a week starting on day 0 for 30 days with survival measured to day 60 post-transplant. Graft survival was assessed by palpation, with 8–20 mice per group from 3–8 replicate experiments.

Figure S2 Total numbers of bulk CD4^+^ conventional T cell (Tconv) and regulatory T cells (Tregs) in the spleen during rejection and tolerance. C57BL/6 recipients were transplanted with heterotopic heart allografts from Act.2W-OVA^+^ BALB/c × C57BL/6 F1 donors. Recipients received anti-CD154 on days 0, 7, and 14 post-transplantation plus donor splenocytes infusion on day 0 (αCD154/DST) or were untreated (No Rx). Recipients were sacrificed on day 7 or day 30 post-transplantation. **(A)** Total CD4^+^ T cells, **(B)** total number of FoxP3^–^CD4^+^ Tconv, and **(C)** total number of FoxP3^+^CD4^+^ Tregs in the spleen. ***p* < 0.01 by one-way ANOVA. Data are presented as mean ± SEM, each point represents one mouse from 4–5 replicate experiments per time point (*n* = 8–13).

Figure S3 Regulatory T cells infiltrate comparably into allografts in rejection and tolerance while conventional T cell infiltration is reduced in tolerance. C57BL/6 recipients were as described in Figure 1. On day 7 or day 30 post-transplantation, mice were sacrificed and their heart grafts were collected, fixed in 10% formalin, and then stained by immunohistochemistry by either CD4 (left panels) or FoxP3 (right panels) staining. One representative panel is shown from each group is shown.

Figure S4 Modest increases in percentages of graft-infiltrating 2W:I-A^b^ CD4^+^ conventional T cells (Tconv) in rejection and 2W:I-A^b^ regulatory T cells (Tregs) in tolerance. C57BL/6 recipients transplanted with heterotopic heart allografts from Act.2W-OVA^+^ BALB/c × C57BL/6 F1 donors, and were untreated (No Rx) or treated with αCD154/DST. Mice were sacrificed on day 7 or day 30 post-transplantation. **(A)** Total number of 2W:I-A^b^(2W)-specific CD4^+^ T cells, **(B)**.percentage of 2W-specific Tconv among total CD4^+^FoxP3^−^ Tconv, and **(C)** percentage of 2W-specific Tregs among total CD4^+^FoxP3^+^ Tregs infiltrating the graft. ****p* < 0.001 by one-way ANOVA. Data are presented as mean ± SEM, and each point represents one mouse from 7–8 replicate experiments (*n* = 12–13).

Figure S5 Phenotypic analysis of bulk regulatory T cells (Tregs) and conventional T cells isolated from recipients with rejecting and tolerant allografts at day 7 post-transplantation. C57BL/6 recipients transplanted with heterotopic heart allografts from Act.2W-OVA^+^ BALB/c × C57BL/6 F1 donors, were untreated (No Rx) or treated with αCD154/DST, and sacrificed on day 7 post-transplantation. Percentage of cells positive (top), and mean fluorescent intensity (bottom) relative to naïve CD4^+^ FoxP3^+^ or FoxP3^−^ cells of **(A,B)**, Helios; **(C,D)**, Neuropilin-1; **(E,F)**, CD25; and **(G,H)**, CD73; in naïve, acutely rejecting (No Rx), and αCD154/DST-treated (+Rx) mice. **p* < 0.05, ***p* < 0.01, ****p* < 0.001 by two-way ANOVA. Mean ± SEM is shown, and each point represents one animal from three replicate experiments (*n* = 4–6/group).

Figure S6 Analysis of Ki67 expression on 2W:I-A^b^-specific conventional T cell (Tconv) isolated from recipients with rejecting and tolerant allografts at day 7 post-transplantation. C57BL/6 recipients transplanted with heterotopic heart allografts from Act.2W-OVA^+^ BALB/c × C57BL/6 F1 donors, were untreated (No Rx) or treated with αCD154/DST, and sacrificed on day 7 post-transplantation. **(A)** Sample gating strategies for Ki67 expression by 2W:I-A^b^-specific Tconvs. **(B)** Percent of 2W:I-A^b^-specific Tconvs expressing Ki67. **p* < 0.05, ****p* < 0.001 by one-way ANOVA. Mean ± SEM is shown, and each point represents one animal from four replicate experiments (*n* = 5–10/group).
