# Supplementary figures and images for: Equal Expansion of Endogenous Transplant-Specific Regulatory T Cell and Recruitment Into the Allograft During Rejection and Tolerance
Source: Front Immunol. 2018 Jun 20;9:1385. doi: 10.3389/fimmu.2018.01385 (PMC6020780; doi:10.3389/fimmu.2018.01385)

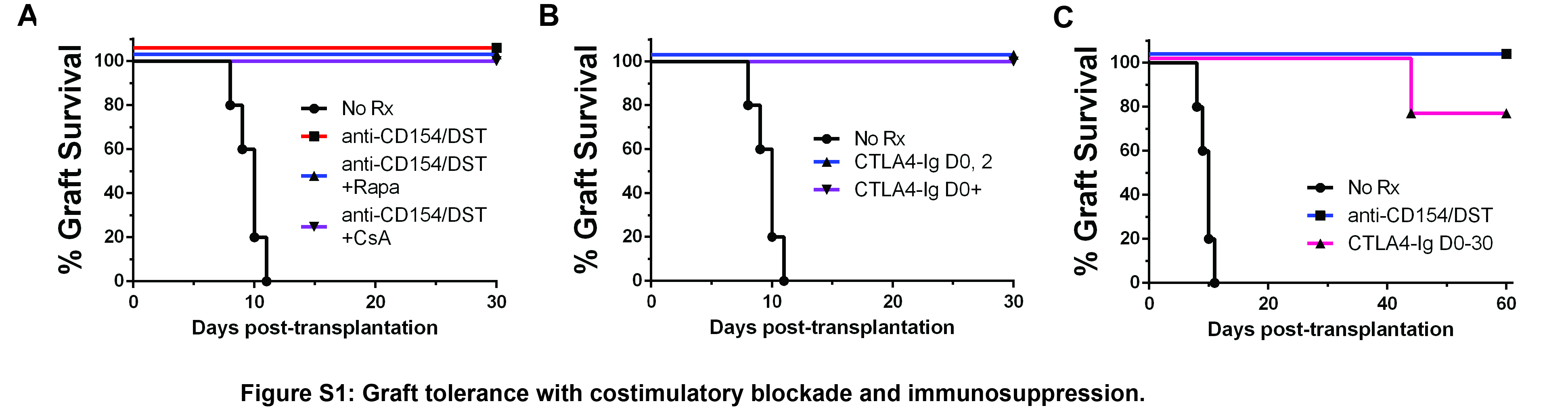

Supplement: Figure S1 — Graft survival with costimulatory blockade and immunosuppression. Act.2W-OVA+ C57BL/6-BALB/c F1 hearts were transplanted into wild-type C57BL/6 recipients: (A) with no treatment (No Rx), or anti-CD154 on day 0, 7, and 14 with DST on day 0 (αCD154/DST) alone or in combination with daily intraperitoneal injections of rapamycin or cyclosporine A for 30 days. (B) With no treatment (No Rx), or CTLA4-Ig on days 0 and 2 post-transplant or twice a week starting on day 0 for 30 days, (C) with anti-CD154 on day 0, 7, and 14 with DST on day 0 or CTLA4-Ig twice a week starting on day 0 for 30 days with survival measured to day 60 post-transplant. Graft survival was assessed by palpation, with 8–20 mice per group from 3–8 replicate experiments. [file image_1.TIF]

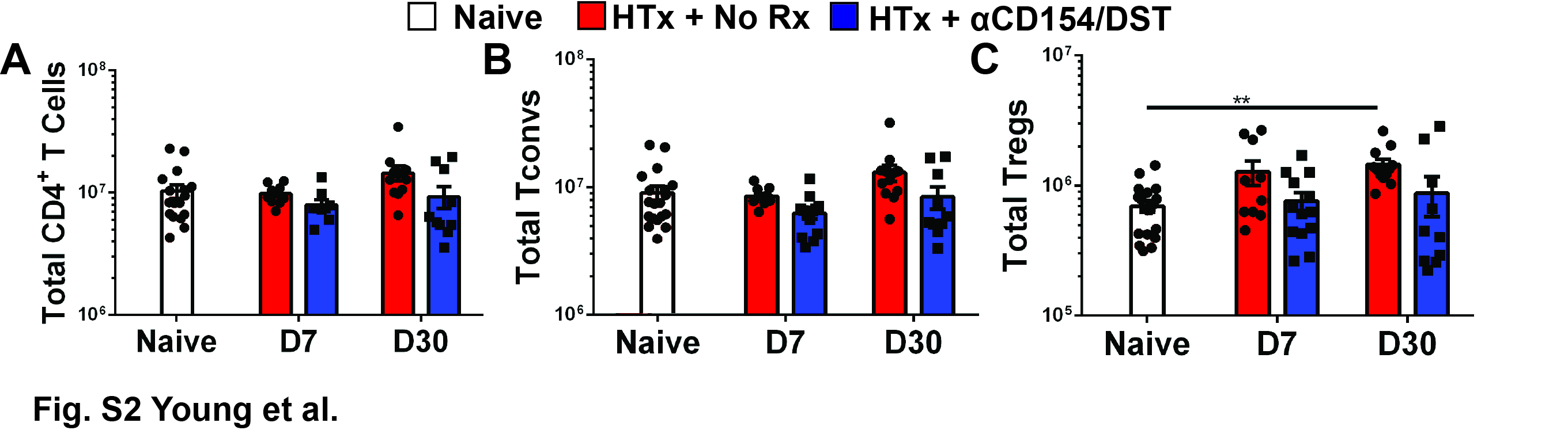

Supplement: Figure S2 — Total numbers of bulk CD4+ conventional T cell (Tconv) and regulatory T cells (Tregs) in the spleen during rejection and tolerance. C57BL/6 recipients were transplanted with heterotopic heart allografts from Act.2W-OVA+ BALB/c × C57BL/6 F1 donors. Recipients received anti-CD154 on days 0, 7, and 14 post-transplantation plus donor splenocytes infusion on day 0 (αCD154/DST) or were untreated (No Rx). Recipients were sacrificed on day 7 or day 30 post-transplantation. (A) Total CD4+ T cells, (B) total number of FoxP3–CD4+ Tconv, and (C) total number of FoxP3+CD4+ Tregs in the spleen. **p < 0.01 by one-way ANOVA. Data are presented as mean ± SEM, each point represents one mouse from 4–5 replicate experiments per time point (n = 8–13). [file image_2.TIF]

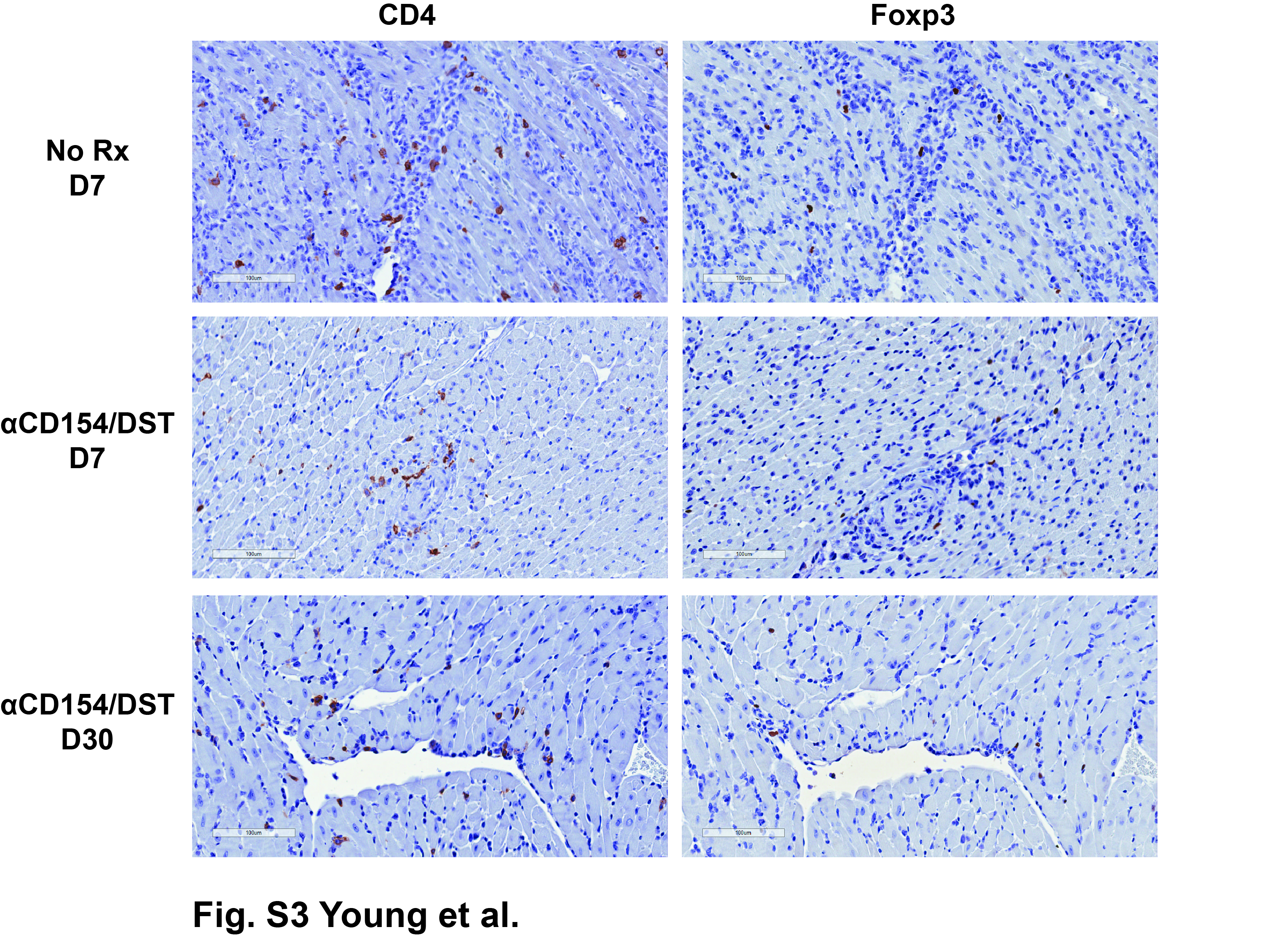

Supplement: Figure S3 — Regulatory T cells infiltrate comparably into allografts in rejection and tolerance while conventional T cell infiltration is reduced in tolerance. C57BL/6 recipients were as described in Figure 1. On day 7 or day 30 post-transplantation, mice were sacrificed and their heart grafts were collected, fixed in 10% formalin, and then stained by immunohistochemistry by either CD4 (left panels) or FoxP3 (right panels) staining. One representative panel is shown from each group is shown. [file image_3.TIF]

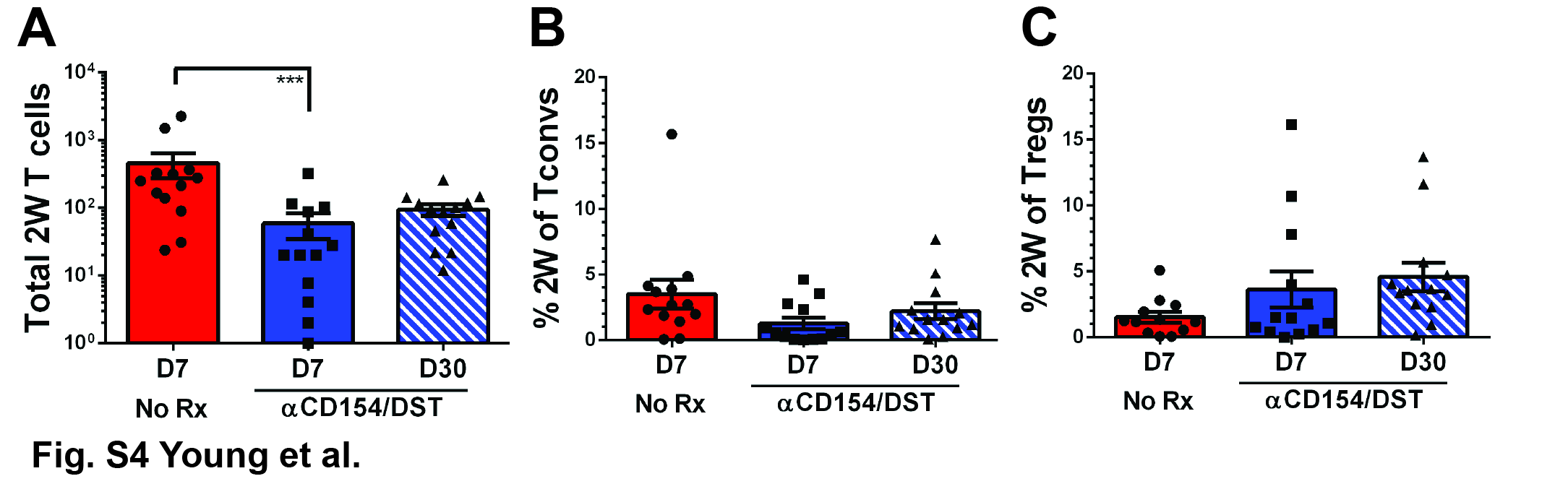

Supplement: Figure S4 — Modest increases in percentages of graft-infiltrating 2W:I-Ab CD4+ conventional T cells (Tconv) in rejection and 2W:I-Ab regulatory T cells (Tregs) in tolerance. C57BL/6 recipients transplanted with heterotopic heart allografts from Act.2W-OVA+ BALB/c × C57BL/6 F1 donors, and were untreated (No Rx) or treated with αCD154/DST. Mice were sacrificed on day 7 or day 30 post-transplantation. (A) Total number of 2W:I-Ab(2W)-specific CD4+ T cells, (B).percentage of 2W-specific Tconv among total CD4+FoxP3− Tconv, and (C) percentage of 2W-specific Tregs among total CD4+FoxP3+ Tregs infiltrating the graft. ***p < 0.001 by one-way ANOVA. Data are presented as mean ± SEM, and each point represents one mouse from 7–8 replicate experiments (n = 12–13). [file image_4.TIF]

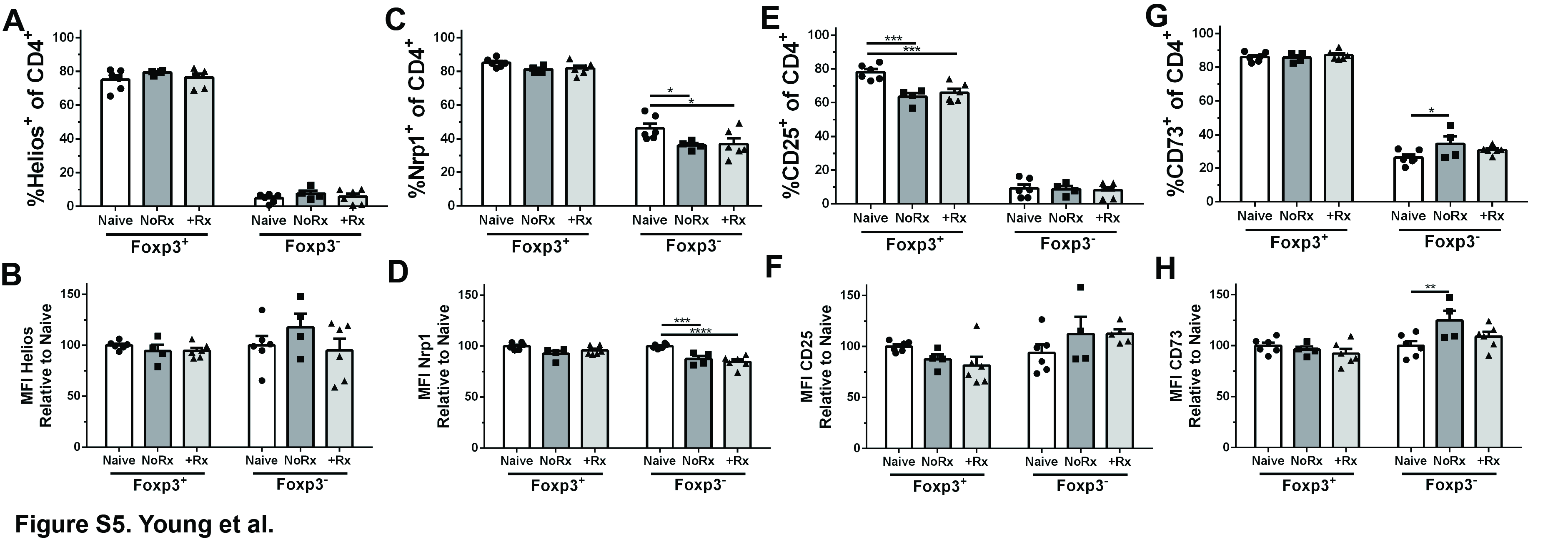

Supplement: Figure S5 — Phenotypic analysis of bulk regulatory T cells (Tregs) and conventional T cells isolated from recipients with rejecting and tolerant allografts at day 7 post-transplantation. C57BL/6 recipients transplanted with heterotopic heart allografts from Act.2W-OVA+ BALB/c × C57BL/6 F1 donors, were untreated (No Rx) or treated with αCD154/DST, and sacrificed on day 7 post-transplantation. Percentage of cells positive (top), and mean fluorescent intensity (bottom) relative to naïve CD4+ FoxP3+ or FoxP3− cells of (A,B), Helios; (C,D), Neuropilin-1; (E,F), CD25; and (G,H), CD73; in naïve, acutely rejecting (No Rx), and αCD154/DST-treated (+Rx) mice. *p < 0.05, **p < 0.01, ***p < 0.001 by two-way ANOVA. Mean ± SEM is shown, and each point represents one animal from three replicate experiments (n = 4–6/group). [file image_5.TIF]

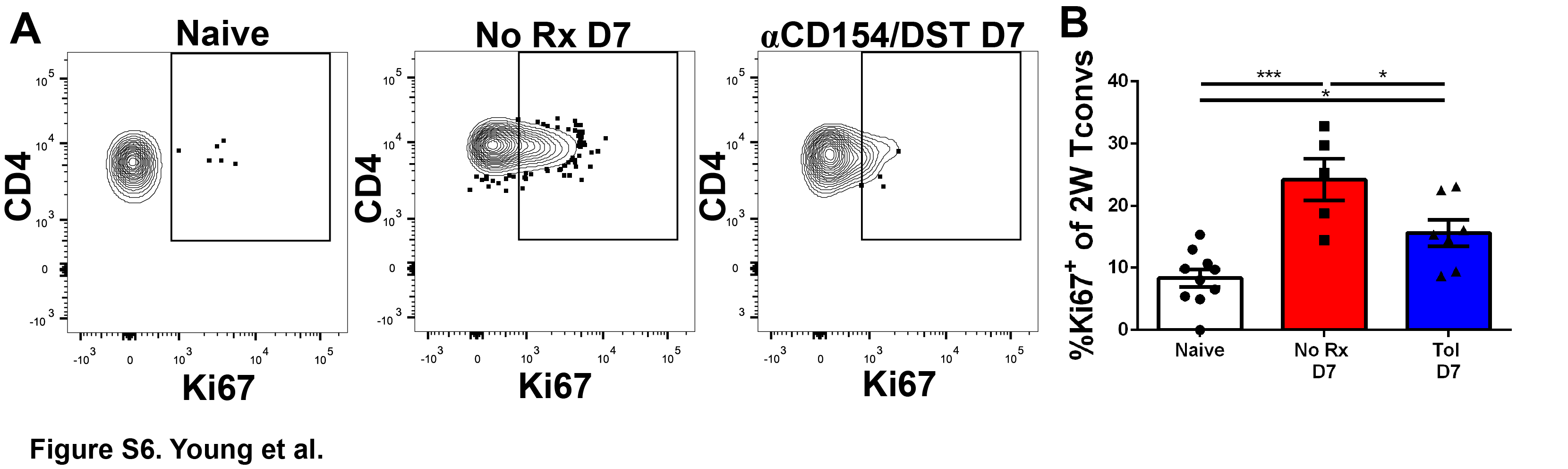

Supplement: Figure S6 — Analysis of Ki67 expression on 2W:I-Ab-specific conventional T cell (Tconv) isolated from recipients with rejecting and tolerant allografts at day 7 post-transplantation. C57BL/6 recipients transplanted with heterotopic heart allografts from Act.2W-OVA+ BALB/c × C57BL/6 F1 donors, were untreated (No Rx) or treated with αCD154/DST, and sacrificed on day 7 post-transplantation. (A) Sample gating strategies for Ki67 expression by 2W:I-Ab-specific Tconvs. (B) Percent of 2W:I-Ab-specific Tconvs expressing Ki67. *p < 0.05, ***p < 0.001 by one-way ANOVA. Mean ± SEM is shown, and each point represents one animal from four replicate experiments (n = 5–10/group). [file image_6.TIF]
